# The costs and benefits of a prison needle and syringe program in Australia, 2025–30: a modelling study

**DOI:** 10.5694/mja2.52640

**Published:** 2025-03-24

**Authors:** Farah Houdroge, Samantha Colledge‐Frisby, Nadine Kronfli, Rebecca J Winter, Joanne Carson, Mark Stoove, Nick Scott

**Affiliations:** ^1^ The Burnet Institute Melbourne VIC; ^2^ National Drug Research Institute Perth WA; ^3^ McGill University Health Centre Montreal Canada; ^4^ Centre for Outcomes Research and Evaluation Research, Institute of the McGill University Health Centre Montreal Canada; ^5^ St Vincent's Hospital Melbourne Melbourne VIC; ^6^ The Kirby Institute, University of New South Wales Sydney NSW; ^7^ Monash University Melbourne VIC

**Keywords:** Prison, Cost‐benefit analysis, Hepatitis, viral, Substance abuse, intravenous

## Abstract

**Objectives:**

To estimate and compare the costs and benefits of introducing a prison needle and syringe program in all Australian prisons.

**Study design:**

Stochastic compartmental modelling study.

**Setting:**

All Australian prisons, 1 January 2010 to 31 December 2030.

**Intervention:**

Introduction of a prison needle and syringe program in all Australian prisons during 1 January 2025 – 1 January 2027, with the aim of covering 50% of people who inject drugs in prison by 1 January 2030.

**Main outcome measures:**

Projected new hepatitis C virus (HCV) infections and hospitalisations with injection‐related bacterial and fungal infections in prisons, with and without the needle and syringe program; costs of the program; savings in treatment costs for HCV and injection‐related bacterial and fungal infections; benefit–cost ratio of the program.

**Results:**

In the base scenario (no prison needle and syringe program), the projected number of new HCV infections during 2025–2030 was 2932 (uncertainty interval [UI], 2394–3507) and the projected number of hospitalisations with injection‐related bacterial and fungal infections was 3110 (UI, 2596–3654). With the prison needle and syringe program, it was projected that 894 (UI 880–912) new HCV infections (30%; UI, 26–37%) and 522 (UI, 509–532) hospitalisations with injection‐related bacterial and fungal infections (17%; UI, 15–20%) would be averted; the incidence of new HCV infections would be reduced from 3.1 (UI, 2.5–3.7) to 1.3 (UI, 1.0–1.7) per 100 person‐years among people who inject drugs in prison. The estimated cost of the program was $12.2 million (UI, $7.6–22.2 million), and the saved care costs for HCV and injection‐related infections were $31.7 million (UI, $29.3–34.6 million), yielding a benefit–cost ratio of 2.6 (UI, 1.4–4.1). The benefit–cost ratio was also greater than one for scenarios in which the assumptions and base values for several parameters were varied.

**Conclusions:**

Each dollar spent on a needle and syringe program in Australian prisons could save $2.60 in treatment costs for HCV and other injection‐related infections.

**The known**: Community‐based needle and syringe programs for people who inject drugs in Australia are cost‐effective, but the potential benefits of prison programs have not been modelled.

**The new**: For each dollar spent on introducing and expanding a needle and syringe program in Australian prisons to provide 50% coverage of people who inject drugs in prison by 1 January 2030, $2.60 in health care costs for people with hepatitis C virus and other injection‐related infections could be saved.

**The implications**: Our modelling findings support the introduction of a prison needle and syringe program across Australia to help achieve WHO hepatitis C virus elimination targets.

The Australian government has committed to meeting the 2030 hepatitis C virus (HCV) elimination targets set by the World Health Organization.^1^, ^2^ These targets include reducing the annual number of new HCV infections to fewer than two per 100 people who inject drugs, and providing annual needle and syringe program coverage of 300 needles and syringes per person who injects drugs. Needle and syringe programs are internationally endorsed, evidence‐based public health interventions that reduce the spread of bloodborne viruses and the incidence of injection‐related infections.^3^, ^4^, ^5^

Australia is one of ten countries classified as having high needle and syringe program coverage, and the estimated number of needles and syringes distributed per person who injects drugs is the highest in the world.^6^ In addition, the introduction of subsidised direct‐acting antiviral medications (DAAs) in March 2016 reduced the prevalence of chronic HCV infections among people who inject drugs in the community from 51% in 2015 to 12% in 2022.^7^ However, recent declines in testing and treatment have jeopardised progress toward HCV elimination.^8^

As the proportion of people with histories of injecting drug use is larger in prisons than in the community, prisons are important sites for HCV testing and treatment interventions.^9^ In 2022, 35% of HCV treatments in Australia were provided in prisons.^8^ The incidence of HCV infections was significantly reduced in New South Wales prisons during 2014–19 by high DAA treatment coverage.^10^ However, among participants who reported injecting drugs during imprisonment, the reduction in the incidence of HCV re‐infection (39%) was lower than that in the incidence of primary infections (64%), and the re‐infection incidence remained high at 9.34 per 100 person‐years.^10^ The impact of DAA treatment on HCV infections in prisons is undermined by the lack of evidence‐based HCV infection prevention services. Effective harm reduction services, such as prison needle and syringe programs and opioid substitution therapy, are crucial for eliminating HCV infections in prisons.^11^ Prison needle and syringe programs are also likely to reduce the incidence of injection‐related bacterial and fungal infections; rates of hospitalisation with these infections are relatively high, despite the substantially lower frequency of injecting in prisons.^12^ Additional benefits include facilitating health‐seeking behaviours, such as testing and entry into substance dependence programs.^3^

In 2023, prison needle and syringe programs were operating in eleven countries, compared with community needle and syringe programs in 93 countries.^13^ There is no prison needle and syringe program in Australia, despite advocacy by the Australian Medical Association^14^ and research and community organisations,^15^, ^16^, ^17^ as well as evidence for the effectiveness and cost‐effectiveness of community needle and syringe programs.^18^, ^19^ The potential health care benefits of needle and syringe programs in Australian prisons and the associated cost savings have not been estimated. We therefore modelled the costs and benefits of introducing prison needle and syringe programs in all Australian prisons.

## Methods

We report our stochastic compartmental model‐based study according to the reporting guideline for economic evaluations of health interventions outlined in the CHEERS 2022 statement.^20^

### Prison model

#### Model overview

We employed a stochastic compartmental model to estimate the impact of prison needle and syringe programs on HCV infections and hospitalisations with injection‐related bacterial or fungal infections in people in Australian prisons. We did not examine human immunodeficiency virus (HIV) infections because their prevalence among people who inject drugs in Australia is low (2.1% in 2022) and HIV treatment coverage is high.^21^ The model population was an aggregate of all 102 Australian prisons, parameterised using Australian Bureau of Statistics data^22^, ^23^ and epidemiological data from cohort study and surveillance reports.^8^, ^10^, ^12^, ^21^ The model was run from 1 January 2010 to 31 December 2030. The modelling methodology is described in detail in the Supporting Information, part 1, which includes the model input data (table 1).

#### Incarceration and release

The model has a time‐varying entry rate, as individuals enter the model according to the annual number of receptions into full time custody, and they are released according to an estimated length of stay in prison that is calibrated to fit the annual incarcerated population count.

#### HCV infections and hospitalisations with other injection‐related infections in prisons

The probabilities of injecting drug use in prison were derived from the SToP‐C study^24^ (Supporting Information, part 2). People in prison are stratified by injecting drug use and HCV RNA status during imprisonment. For people entering prison with a history of injecting drug use, chronic HCV infection probability is derived from time‐varying estimates of community prevalence among people who inject drugs. For people without a history of injecting drug use, chronic HCV infection prevalence is assumed to be constant. The time‐varying probability of cure for people with chronic HCV infections, regardless of their injecting behaviour, is derived from annual numbers of treatments in prisons.^8^ For people who inject drugs and are HCV RNA‐negative, we assumed equal rates of primary infections and re‐infections, calibrated to match the overall incidence of HCV infections among people in prison. The model also assumed a constant risk of hospitalisation with injection‐related bacterial and fungal infections for people who inject drugs.

#### Preventive role of the prison needle and syringe program

We assumed that people who inject drugs who participate continuously in the prison needle and syringe program do not share needles and syringes, and that their risk of HCV infection was consequently zero. We also assumed a 62% reduction in the risk of hospitalisation with injection‐related bacterial and fungal infections among program participants.^25^

#### Proposed model for the prison needle and syringe program

Diverse types of prison needle and syringe programs have been described, including fixed site programs, peer‐based programs, and dispensing machines. We modelled a program based on a one‐for‐one exchange program proposed to the Australian Capital Territory government in 2015 following the 2011 evaluation of drug policy and services at the Alexander Maconochie Centre in Canberra.^26^ This model was informed by overseas best practice guidelines,^27^ the operating conditions of Australian prisons, and similar programs in Europe.^28^ The prison needle and syringe program is open to all people in prison who ask to attend the prison health service. Needle and syringe program kits are provided face‐to‐face by prison health staff, facilitating confidentiality and other health care. Information about needle and syringe program security and safety conditions, as well as about harm reduction and other education, is provided. Exchanging used for new kits also requires a request to visit the health service. Kits include two standard 1 mL syringes, sterile single use cotton filters, disinfectant swabs, sterile water ampoules, intranasal naloxone, a “cooker” for drug preparation, and printed information about health services and drug treatment in prison, safe injecting practices, prevention of bloodborne virus infections, and overdose management.

### Model‐based analysis

#### Scenarios

We projected the total number of new HCV infections and hospitalisation with injection‐related bacterial and fungal infections in prisons during 1 January 2025 – 31 December 2030 in two scenarios:

- base scenario (standard practice): treatment of HCV infections, but no prison needle and syringe program;
- prison needle and syringe program rolled out across all Australian prisons during 1 January 2025 – 1 January 2027; coverage of people who inject drugs in prison increases linearly from zero on 1 January 2025 to 50% on 1 January 2030.

#### Epidemiological outcomes

The primary epidemiological outcomes were the projected number and incidence of new HCV infections, and hospitalisations with injection‐related bacterial and fungal infections in prisons among people who inject drugs. The stochastic model was run five hundred times for each scenario to calculate the median value and 95% uncertainty intervals (UIs; 2.5th–97.5th percentiles) for each outcome. The number of infections or hospitalisations averted by the prison needle and syringe program scenario were calculated using a bootstrap resampling method.

### Cost–benefit analysis

The economic analysis did not separately investigate costs to the public health and correctional systems, as this may differ between jurisdictions; we instead estimated overall costs and benefits.

#### Costs

Costs included initial start‐up costs, ongoing operational expenses, and costs associated with enrolment and subsequent kit exchanges. Start‐up costs covered an initial one‐hour training session for health care staff about needle and syringe program procedures and policies, and assumed 24% annual staff turnover (Australian Bureau of Statistics 2022–23 job mobility report^29^). Annual operational costs included ongoing expenses (eg, staff time to order supplies, and shipping costs). Staff time and resource costs for each participant included costs for the participant's first health service visit for receiving their initial kit, as well as staff time for managing kit exchanges and the cost of the kit components. Costs are reported in 2023 Australian dollars using the Reserve Bank of Australia inflation calculator (https://www.rba.gov.au/calculator), discounted at 5% per year^30^ (Box 1). Further details are included in the Supporting Information, part 3.

Box 1 **Cost components for the modelled prison needle and syringe program in Australia (2023 dollars)**

**Table:** 

Cost component	Purpose	Point estimate (lower and upper bound)
Start‐up costs		
Primary health care nurse (hourly wage)	Program education: one hour per nurse per prison	$57.88 ($52.48–63.33)
Annual operational costs		
Shipping (Australia Post: large box flat rate)	Supplies deliveries: one delivery per month per prison	$59.20
Primary health care nurse (hourly wage)	Ordering of supplies: five minutes per month per prison	$57.88 ($52.48–63.33)
One‐off costs per participant		
Primary health care nurse (hourly wage)	First encounter with participant: 15 minutes	$57.88 ($52.48–63.33)
Prison needle and syringe program kit: case, components, single‐dose intranasal naloxone	Participant provided with their first kit	$32.00
Costs per unit dispensed		
Primary health care nurse (hourly wage)	Kit exchange: 5 minutes	$57.88 ($52.48–63.33)
Prison needle and syringe program kit: components only	Exchanges per participant per month: 4	$4.00
Naloxone: single‐dose intranasal spray	Replacements per one hundred participants per year: twelve	$24.97
Sharps container: four litres	Syringes per container: 125	$9.63

#### Benefits

We estimated the averted costs of treating HCV infections and hospitalisations with injection‐related bacterial and fungal infections. The cost per HCV cure was based on recent Australian study estimates for prisons and the community.^31^, ^32^ The proportion of all treatments of HCV infections that were undertaken in the community (about 43% across simulations) is a direct model outcome influenced by the prevalence of chronic HCV infections in prisons, population size, the number of treatments, and the estimated time in prison. DAA treatment costs were based on Pharmaceutical Benefits Scheme information.^32^ Deriving the cost of treating injection‐related bacterial and fungal infections in Australian public hospitals is described in the Supporting Information, part 4. Benefits are reported in 2023 Australian dollars and discounted at 5% per year (Box 2).

Box 2 **Health care costs for managing hepatitis C virus (HCV) infections and injection‐related bacterial and fungal infections (2023 dollars)**

**Table:** 

Component	Point estimate (lower and upper bounds)
HCV testing in the community^31^	$3754 ($3376–4130)
HCV testing in prison^32^	$1499 ($1499–1527)
HCV infection treatment^33^ ^,^*	$36 111 ($34 018–36 111)
Other injection‐related infection management^†^	$13 809 ($10 595–20 100)

* Based on dispensed pharmaceutical price for maximum quantity of direct‐acting antiviral medication for the treatment of HCV infections. Point estimate: sofosbuvir/velpatasvir, treatment duration of twelve weeks; upper bound: sofosbuvir/velpatasvir/voxilaprevir, treatment duration of twelve weeks; lower bound: glecaprevir/pibrentasvir, treatment duration of eight weeks.^34^ Treatment costs of $5000 and $15 000 were assessed in sensitivity analyses.

† Financial year 2022–23. Sources: Supporting Information, part 4.

#### Benefit–cost ratio

Median estimates of total costs, total benefits, and the benefit–cost ratio were calculated using the median number of HCV infections and hospitalisations with injection‐related bacterial and fungal infections averted and point estimates of costs (with uncertainty intervals) estimated using bootstrapping. A benefit–cost ratio greater than one indicates that the financial benefits of the program exceed its costs.

### Sensitivity analyses

In univariate sensitivity analyses, we examined various levels of prison needle and syringe program coverage, changes in the costs and benefits discount rate, lower and upper bounds for cost and benefit parameters, the proportion of people with a history of injecting drug use, and the incidence and prevalence of HCV infections and hospitalisations with injection‐related bacterial and fungal infections. We also undertook analyses by prison security level (Supporting Information, part 5), and analyses with different assumptions regarding the relationship between program coverage and reduction in infection risks, including rates of receptive sharing of needles and syringes by people using the program, and whether the program was used by people with lower or higher injection risks (Supporting Information, part 6), and how increasing the availability of opioid substitution therapy affected outcomes. To ensure the consistency of the model with empirical data, particularly when changing model input variables, it was recalibrated as required.

### Ethics approval

We did not seek ethics approval for our use of publicly available aggregate data.

## Results

### Effect of the prison needle and syringe program on infections and hospitalisations

In the base scenario (no prison needle and syringe program), the projected number of new HCV infections during 2025–2030 was 2932 (UI, 2394–3507) and the projected number of hospitalisations with injection‐related bacterial and fungal infections was 3110 (UI, 2596–3654). Were a prison needle and syringe program introduced during 2025–27, it was projected that 894 (UI, 880–912) new HCV infections (30%; UI, 26–37%) and 522 (UI, 509–532) hospitalisations with injection‐related bacterial and fungal infections (17%; UI, 15–20%) would be averted; the incidence of HCV infections would be reduced from 3.1 (UI, 2.5–3.7) to 1.3 (UI, 1.0–1.7) per 100 person‐years among people who inject drugs in prison. Without the prison needle and syringe program, none of the simulations reached the WHO HCV incidence target of fewer than two per 100 people who inject drugs per year; with the program, all simulations reached this target (Box 3, Box 4).

Box 3 Projected impact on the incidence of hepatitis C virus (HCV) infections and hospitalisations with injection‐related bacterial and fungal infections of a prison needle and syringe program introduced in all Australian prisons during 1 January 2025 – 1 January 2027, with 50% program coverage by 1 January 2030*

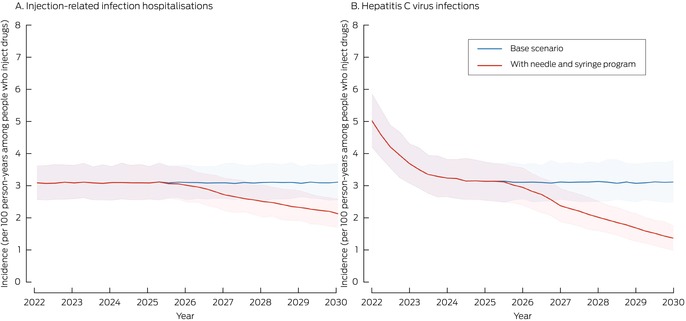

* The curves indicate the medians at quarter‐year intervals from five hundred sampled model trajectories; the shaded areas indicate the 95% range of values (2.5th to 97.5th percentiles) across five hundred runs.

Box 4 Projected outcomes, 2025–2030, of introducing a prison needle and syringe program in all Australian prisons during 1 January 2025 – 1 January 2027, with 50% program coverage by 1 January 2030*

**Table:** 

Outcome	Base scenario	Prison needle and syringe program scenario
Incidence of new HCV infections, 2025–2030 (UI)	2932 (2394–3507)	2036 (1605–2501)
Incidence of injection‐related bacterial and fungal infection hospitalisations, 2025–2030 (UI)	3110 (2596–3654)	2586 (2119–3086)
Incidence of HCV infections per 100 person‐years among people who inject drugs in prison, 2030 (UI)	3.1 (2.5–3.7)	1.3 (1.0–1.7)
Incidence of injection‐related bacterial and fungal infection hospitalisations per 100 person‐years among people who inject drugs in prison, 2030 (UI)	3.1 (2.6–3.6)	2.1 (1.7–2.6)
HCV infections averted, 2025–2030 (UI)	—	894 (880–912)
Injection‐related bacterial and fungal infection hospitalisations averted, 2025–2030 (UI)	—	522 (509–532)
Costs ($ million), 2025–2030 (UI)	—	12.2 (7.6–22.2)
Benefits ($ million), 2025–2030 (UI)	—	31.7 (29.3–34.6)
Benefit–cost ratio, 2025–2030 (UI)	—	2.6 (1.4–4.1)

UI = uncertainty interval.

* UIs for new infections and hospitalisations were derived directly from the five hundred sampled runs. For infections and hospitalisations averted, costs, benefits, and benefit–cost ratio, UIs were computed using the bootstrap method.

### Financial costs and benefits of the prison needle and syringe program

The costs of the prison needle and syringe program during 2025–2030 were projected to be $12.2 million (UI, $7.6–22.2 million); the averted care costs for HCV and injection‐related bacterial and fungal infections were $31.7 million (UI, $29.3–34.6 million), yielding a benefit–cost ratio of 2.6 (UI, 1.4–4.1). Costs for activities related to kit distribution constituted 82% (UI, 73–90%) of total expenditure (including 44% for health care staff time and 36% for the kits), participant enrolment costs constituted 14% (UI, 8–22%), annual operational expenses 3% (UI, 1–4%), and start‐up expenses 1% (UI, 1–1%). Averted HCV infection care costs comprised 83% (UI, 76–85%) of cost savings (Box 5).

Box 5 Costs and benefits, 2025–2030, of introducing a prison needle and syringe program in all Australian prisons during 1 January 2025 – 1 January 2027, with 50% program coverage by 1 January 2030*

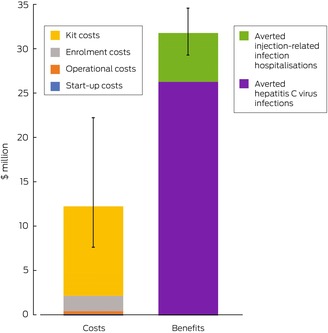

* 2023 Australian dollars.

### Sensitivity analyses

The benefit–cost ratio of the prison needle and syringe program exceeded one for all scenarios examined, except when the cost of HCV treatment was reduced from $36 111 to $5000 per course (ratio, 0.9), or the prevalence of chronic HCV infections among people with histories of injecting drug use was 3% (ratio, 0.9) (Box 6). If the proportion of people who inject drugs who continue to inject in prison was reduced by 20% or 60% from 2025 onwards by expanded access to opioid substitution treatment, the benefit–cost ratio declined from 2.6 to respectively 2.5 or 2.2; the ratio was still greater than one if 5% (ratio, 2.5) or 19% of people who participated in the program (ratio, 2.1) continued to share injection equipment (Supporting Information, table 3). The benefit–cost ratio was most sensitive to the initial incidence of HCV infections; reducing it by 50% (from 21.7 to 10.9 per 100 person‐years among people who inject drugs in prison) resulted in a 50% reduction in the benefit–cost ratio (1.3); doubling it (to 43.5 per 100 person‐years) tripled the benefit–cost ratio (7.7). Other parameters that markedly influenced the benefit–cost ratio were the prevalence of chronic HCV infections among people with injecting drug use histories (6%: ratio, 1.4; 24%: ratio, 6.7), and the proportion of people with histories of injecting drug use (28%: ratio, 2.2; 100%: ratio, 3.9) (Box 6).

Box 6 Impact of key parameters on the benefit–cost ratio of the prison needle and syringe program in Australian prisons of key factors: sensitivity analyses*

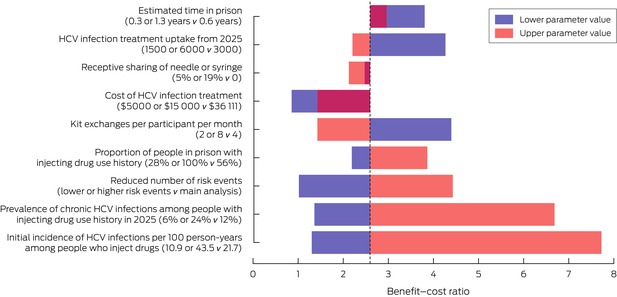

HCV = hepatitis C virus.

* Comparisons with the base scenario. The dotted line indicates the benefit–cost ratio for the main model (2.6). When the benefit–cost ratios for both the lower and upper parameter values lie on the same side of this value, the overlapping area is maroon. The numbers depicted in this graph, and benefit–cost ratios for further sensitivity analyses, are included in the Supporting Information, table 3.

The benefit–cost ratio of implementing the needle and syringe programs in maximum security prisons only was 2.7 (Supporting Information, table 4).

For a hypothetical non‐linear relationship between program coverage and outcomes, the program was cost‐neutral at 50% coverage if the program is disproportionately used by people with lower injecting risks (benefit–cost ratio of 1.0); if disproportionately used by people with higher injecting risks, the benefit–cost ratio was 4.4 (Supporting Information, table 3 and figures 5 and 6).

## Discussion

We estimate that each dollar spent on a needle and syringe program in Australian prisons would save $2.60 in HCV and injection‐related bacterial and fungal infection treatment costs by the end of 2030. We projected that 30% of new HCV infections in prisons and 17% of injection‐related bacterial and fungal infection hospitalisations could be averted during 2025–2030. The incidence of new HCV infections could be reduced to 1.3 (UI, 1.0–1.7) cases per 100 person‐years who inject drugs in prison by 2030, better than the WHO target.

The total estimated cost of the prison needle and syringe program during 2025–2030 was $12.2 million (UI, $7.6–22.2 million); Australian governments spent $27 million per year on community‐based needle and syringe programs during 2000–2009.^20^ About 83% of the projected $31.7 million (UI, $29.3–34.6 million) in cost savings were attributed to averted HCV infections. However, the severe complications of other injection‐related infections impose a considerable burden on health care; the number of hospitalisations with injection‐related infections is increasing in Victoria and New South Wales.^35^, ^36^ Prison needle and syringe programs typically focus on preventing bloodborne virus infections, but their role in reducing the incidence of other injection‐related infections is also important.

Our findings regarding the cost‐effectiveness of a prison needle and syringe program are consistent with those of a mathematical modelling study of community‐based programs in Australia, which found benefit–cost ratios of 1.3 to 5.5 for needle and syringe programs during 2000–2010;^18^ a retrospective economic analysis estimated that for every dollar spent on needle and syringe programs during 2000–2009, more than four dollars were saved in health care costs for people with HIV and HCV infections.^19^ Ours is the first published study to estimate the benefit–cost ratio of prison needle and syringe programs in Australia, and the second study of its type overall.^37^

The risk of HCV transmission is especially high in prisons, as evidenced by the 2019 outbreak in a Queensland facility despite prior local HCV elimination being achieved by DAA treatment.^38^ This outbreak highlights the need to combine increased DAA access with effective harm reduction strategies, including prison needle and syringe programs and opioid substitution therapy, to eliminate HCV infections in prisons.^11^ Despite strong evidence for their safety and efficacy,^3^, ^4^ needle and syringe programs have not been introduced in Australian prisons, a critical gap in the national harm reduction policy that compromises efforts to achieve HCV elimination targets and reduces the value of substantial government spending on DAAs.

In our model, about 44% of the costs of the needle and syringe program were attributed to the time health care staff spent exchanging used injecting equipment, and 36% was associated with program supplies. Hybrid or multimodal program models that include both health services and automated systems (syringe dispensing machines) could be more efficient. Our sensitivity analyses indicated that prison needle and syringe programs are likely to be cost‐effective across a wide range of conditions, particularly in settings where HCV infection incidence, prevalence, and treatment costs are higher than in Australia.

Advocates have long campaigned for prison needle and syringe programs in Australia on the basis of human rights and health equity.^15^, ^16^, ^17^ Australia is a signatory to the United Nations *Standard Minimum Rules for the Treatment of Prisoners* (the Nelson Mandela rules),^39^ which include the responsibility of the state for providing health care in prisons similar to that available in the community. The Australian Medical Association also endorsed needle and syringe programs in their position statement on bloodborne virus infections.^14^ Our study adds economic reasons to the human rights justification, providing an additional rationale for prison needle and syringe programs.

### Limitations

First, we assumed uniform parameters and intervention approaches for all prisons because we did not have epidemiological and behavioural data for individual prisons. Our projections assumed constant parameters (such as the incidence of HCV and other injection‐related infections), but the model did not take variations between jurisdictions into account. Second, our model did not consider changes in HCV infection risk after treatment or reincarceration, oversimplifying HCV transmission patterns in prisons. Further, the main analysis assumed a linear relationship between program coverage and risk reduction; the influence of equipment sharing was explored only in sensitivity analyses. Third, we may have underestimated savings for treating injection‐related bacterial and fungal infections, as we excluded low level infections treated in the prison or in emergency departments, and the effectiveness of needle and syringe programs for averting severe infections is unclear. Fourth, opioid substitution therapy in prisons was not explicitly considered by our model. However, the base scenario was calibrated using recent incidence and prevalence values, which would incidentally reflect opioid substitution treatment coverage. Fifth, our cost estimates assume that the needle and syringe program is implemented as a health intervention, and we included costs for training health staff but not the education of prison officers (the costs of which are unknown and would depend on implementation factors). Finally, focusing on HCV infections and injection‐related bacterial and fungal infection hospitalisations does not consider the benefit of prison needle and syringe programs for preventing different HCV infection states (particularly more advanced disease), HIV and hepatitis B virus infections, and overdoses.

### Conclusions

We estimated that each dollar spent on a needle and syringe program in Australian prisons could save $2.60 in HCV and other injection‐related infection treatment costs. These interventions should be priorities for governments, policymakers, health care providers, and correctional facility administrators to reduce the potential waste in health care spending on treating HCV infections without concurrently supporting prevention measures.

## Competing interests

Nadine Kronfli has received research funding from Gilead Sciences, AbbVie, and ViiV Healthcare, advisory fees from Gilead Sciences, ViiV Healthcare, Merck, and AbbVie, and speaker fees from Gilead Sciences, AbbVie, and Merck, all unrelated to this study. Mark Stoové has received investigator‐initiated research funding from Gilead Sciences and AbbVie and consultant fees from Gilead Sciences for activities unrelated to this study. Rebecca Winter has received investigator‐initiated research funding from Gilead Sciences unrelated to this study.

## Data sharing

This study did not generate original data.

## Supporting information


Supplementary methods and results
