# Supplementary material for: The costs and benefits of a prison needle and syringe program in Australia, 2025–30: a modelling study
Source: Med J Aust. 2025 Mar 24;222(8):396–402. doi: 10.5694/mja2.52640 (PMC12050243; doi:10.5694/mja2.52640)
Supplement: Supplementary file 1 — Supplementary methods and results [file MJA2-222-396-s001.pdf]

MJA
The Medical Journal of Australia

Supporting Information

Supplementary methods and results

This appendix was part of the submitted manuscript and has been peer reviewed. It is posted as supplied by the authors.

Appendix to: Houdroge F, Colledge-Frisby S, Kronfli N, et al. The costs and benefits of a prison needle and syringe program in Australia, 2025–30: a modelling study. *Med J Aust* 2025; doi: 10.5694/mja2.52640.

Supplementary methods

1. Modelling methodology

Figure 1. Schematic representation of the compartmental stochastic model for a prison needle and syringe program. Individuals enter the model based on the yearly number of receptions into full time custody and are classified according to their injecting drug use behaviour in prison and hepatitis C virus (HCV) RNA status (blue arrows). Transitions are described as probabilities of becoming infected with HCV, being treated for HCV infection, or experiencing an injection-related bacterial or fungal infection (black arrows). Individuals exit the model according to a calibrated estimated time spent in prison (dashed arrows). The prison needle and syringe program reduces the probabilities of people who inject drugs in prison acquiring HCV or an injection-related bacterial or fungal infection (red arrows).

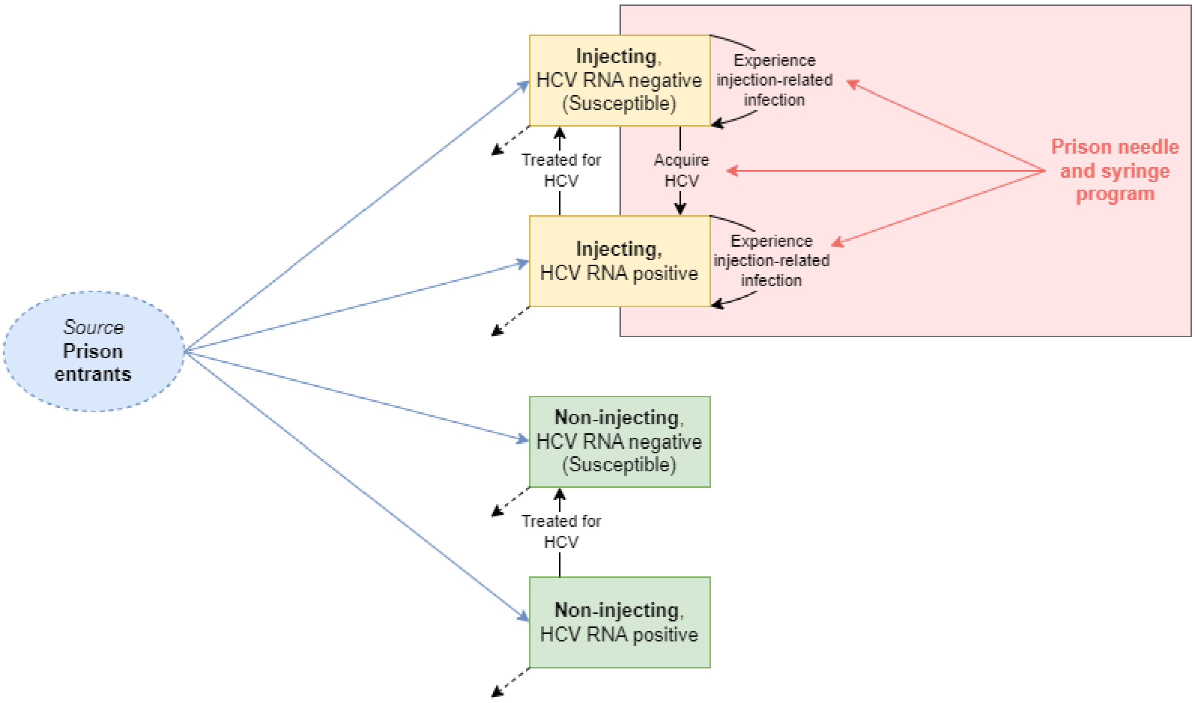

Description and input data

We developed a stochastic compartmental model that incorporates the entire population of people housed in Australian prisons to estimate the number of hepatitis C (HCV) infections and injection-related bacterial or fungal infections prevented in different prison needle and syringe program scenarios. The stochastic model was run 500 times for each scenario, with a time step of one-quarter of a year.

In the model, individuals are introduced through incarceration and exit via release. In prison, they are stratified by their injecting drug use status in prison and their HCV RNA status. Individuals who are HCV RNA-positive and inject drugs in prison can transmit the virus to HCV RNA-negative people who inject drugs in prison. The model treats the rate of HCV re-infection after treatment as equivalent to the rate of primary infection. It also assumes that all individuals who inject drugs are susceptible to injection-related infections.

Incarceration and release

Individuals enter the model according to the annual number of receptions into full time custody (1), and are released according to an estimated length of stay in prison that is calibrated to fit the annual incarcerated population count (2).

Injecting drug use in prison

Of those entering the prison system (prison entrants), 56% report a history of injecting drug use according to data from the SToP-C study, a prospective cohort study in New South Wales prisons (3). In prisons, individuals with a history of injecting drug use were estimated to have a 0.66 probability of continuing to inject in prison,

while those with no injecting drug use history were estimated to have a 0.04 probability of initiating injecting drug use in prison. These probabilities were derived using data from the SToP-C study, using the methodology detailed in part 2 of this file.

Hepatitis C infections in prison

Individuals with a history of injecting drug use entering prison have a time-varying probability of having chronic HCV infections (i.e., being HCV RNA-positive), derived from annual surveillance reports of hepatitis C in Australia (4). The prevalence of chronic HCV infections for individuals without a history of injecting drug use is assumed to be constant at 1.0% according to the National Prison Entrants' Bloodborne Virus and Risk Behaviour Survey Report (5). In prisons, using data from the SToP-C study (3), the model captured HCV infection prevalence among the entire incarcerated population and HCV infection incidence. Annual HCV infection treatment numbers in prisons were based on national reports on progress towards hepatitis C elimination in Australia (6) and a modelling study (7).

People with chronic HCV infections have a time-varying probability of being cured ($prob_{TX}$), assumed to be the same for those who inject or do not inject drugs in prison. Susceptible individuals who inject drugs in prison have a probability of infection ($prob_{INF}$) that is proportional to HCV RNA prevalence (in the subset of people who inject drugs in prison) and the prison needle and syringe program coverage.

Each time step, the number of susceptible individuals who become infected in prison and the number of people with HCV infections who are treated are drawn from binomial distributions with the probabilities:

$$prob_{INF} = \alpha \times prevalence \times \left(1 - NSP_{coverage}\right)$$
$$prob_{TX} = \beta$$

$NSP_{coverage}$ is the proportion of people who inject drugs in prison enrolled in the prison needle and syringe program (time-varying and scenario-dependent), α is a constant calibrated to fit estimates of HCV RNA incidence in prison in base scenario, and β a time-varying treatment rate calculated by dividing the total number of treatments in prison by the number of HCV RNA-positive individuals. It is assumed that the prison needle and syringe program eliminates the risk of needle sharing for people who are actively using it, and that their risk of acquiring HCV infections becomes zero. However, as it is a compartmental model, this does not track individual people, just a time-varying coverage representing the proportion of people who are protected.

Hospitalisations with injection-related bacterial or fungal infections in prison

Each time step, a number of incarcerated individuals who engage in injecting drug use will be admitted to hospital with injection-related infections. This risk can be reduced by the prison needle and syringe program but not eliminated, and is drawn from a binomial distribution with probability $prob_{IRI}$:

$$prob_{IRI} = \gamma \times \left(1 - NSP_{coverage} \times \delta\right)$$

γ is the incidence rate of hospital admissions with injection-related infections in prisons based on data from an Australian study (8). The odds of a skin and soft tissue infection are lower for people who inject drugs if their uptake of the needle and syringe program is high and they undergo opiate substitution treatment (adjusted odds ratio, 0.614; 95% confidence interval, 0.458–0.823) (9). The parameter δ, an efficacy parameter that is the threshold for intervention effectiveness, is derived from this odds ratio. The OR was converted to a relative risk using the formula (10):

$$\delta = \frac{OR}{1 - \gamma(1 - OR)}$$

giving a value of 0.62. This means that, for an individual who engages in injecting drug use and is enrolled in the prison needle and syringe program, the prison needle and syringe program reduces their risk of hospitalisation with an injection-related infection by 62%.

Calibration

The calibration process takes a maximum likelihood approach. Calibration is automated, using a script designed to optimise two parameters in the model: the estimated time spent in prison, determined by fitting to the prison

population size, and parameter α, fitted to HCV infection incidence data (figure 2). The *scipy.optimize* package is used to perform this optimisation; its minimising function reduces the distance between the empirical data and median model predictions. The optimisation resulted in an estimated time spent in prison of 0.63 years (seven and one-half months) and a value for α of 0.53.

Figure 2. Model calibration in the base scenario. The blue lines depict the median model predictions from 500 sampled runs and the shaded areas the central 95th percentile. The empirical data are indicated by the black circles. (a) Yearly incarcerated population. (b) Incidence of injection-related bacterial or fungal infections per 100 people who inject drugs in prison per year. (c) Chronic HCV infection prevalence among the entire incarcerated population. (d) Incidence of HCV infections per 100 people who inject drugs in prison per year.

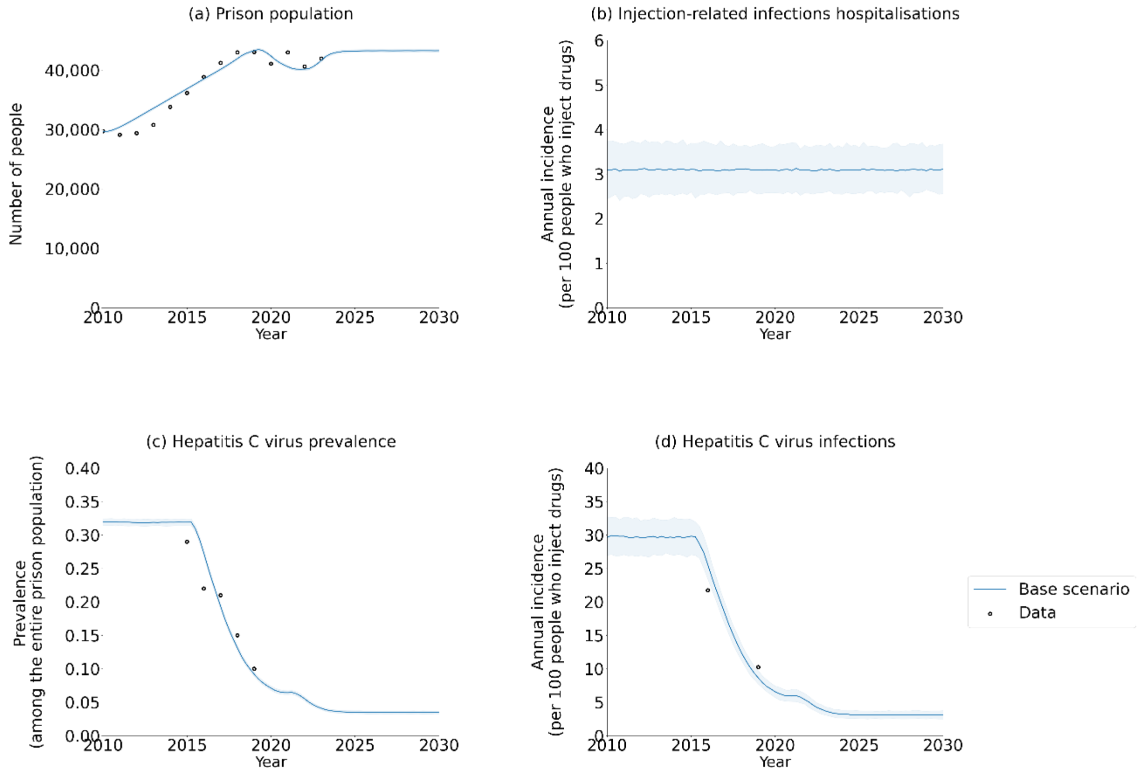

Convergence checks

Convergence checks were performed to determine the time step and sample size required for convergence, using qualitative (visual) and quantitative methods.

Time step

For a sample size of 1000 runs in the base scenario, the step size was varied from 0.01 to one year in increments of 0.05 years, and we assessed the goodness of fit (how well the median fit the data) and stability of the results (oscillatory behaviour of the median and interval width) by visual examination of outcomes. We also analysed the consistency of outcomes over consecutive time steps by checking changes in the median outcomes of total HCV and injection-related bacterial or fungal infection incidence during 2018–2030. We found that larger time steps (≥ 0.3 years) were unsuitable because of large variations in incidence over consecutive time steps, suggesting that the model might not be capturing disease transmission dynamics efficiently, while smaller time steps (≤ 0.15 years) led to significant oscillations in outcomes and wide confidence intervals, suggesting instability (figure 3). Time steps of between 0.15 and 0.3 years yielded the most consistent and stable results, leading us to select 0.25 years as the optimal time step.

Figure 3. (A) Time step sensitivity. Plot showing the incidence of new HCV infections and injection-related bacterial or fungal infections during 2025-2030 across varying time step sizes. The median outcomes of the 1000 runs are indicated by the circles, and their range by the bars. Shaded regions indicate unstable (grey area at ≤ 0.15 years) and invalid results (grey area at ≥ 0.3 years). (B-D) Outcomes evolution with different time steps.

(A) Cumulative outcomes, 2025-2030

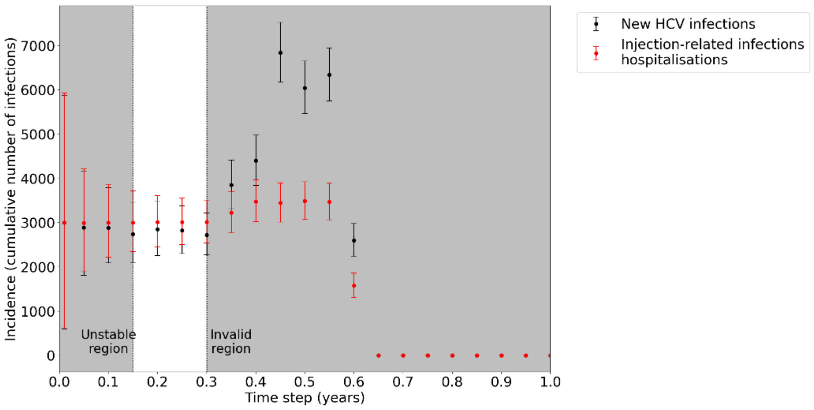

(B) dt = 0.01 years

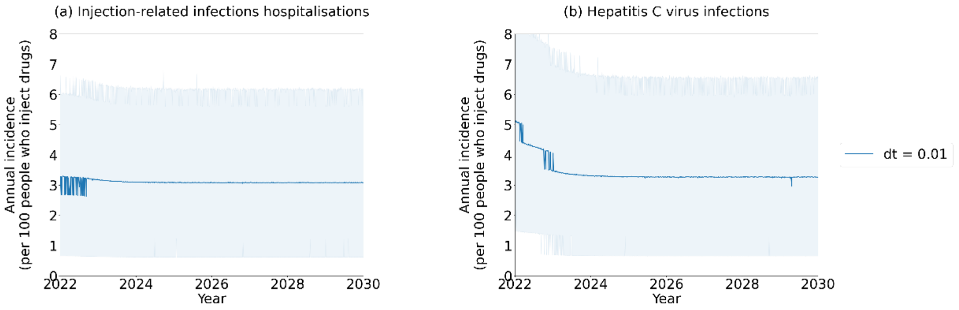

(C) dt = 0.1 years

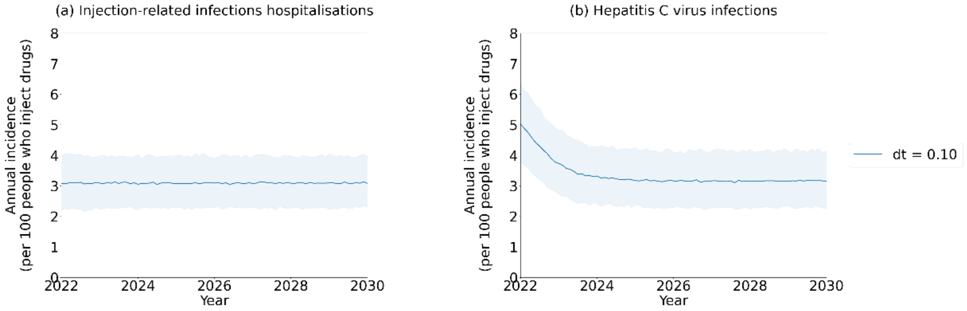

(D) dt = 0.25 years

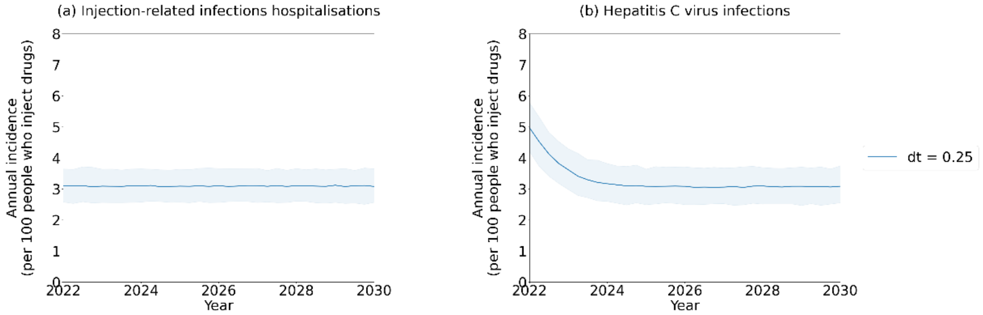

Number of stochastic runs

With a fixed step size of 0.25 years, the sample size was varied from 50 to 1000 runs in the base scenario, increasing in increments of 50. We assessed the stability of the total median HCV and injection-related bacterial or fungal infections during the period with different sample sizes, the smoothness of the results, and the behaviour of the oscillations in the medians and uncertainty intervals over time. The stability of the median total outcomes and the width of the confidence interval were consistent across the range, indicating the robustness in model predictions at lower sample sizes. Oscillations of the median stabilised beyond a sample size of 400 runs, but were not problematic at lower sample sizes (Figure 4). A sample size of 500 runs was selected.

Figure 4. (A) Total median outcomes. Plot showing the incidence of new HCV and injection-related bacterial or fungal infections over 2025-2030 with different sample sizes (number of runs). The median outcomes are indicated by the circles, and their range by the error bars. (B-D) Outcomes evolution with different sample sizes. The incidence of HCV and other injection-related infections are plotted against time.

(A) Cumulative outcomes over 2018-2030

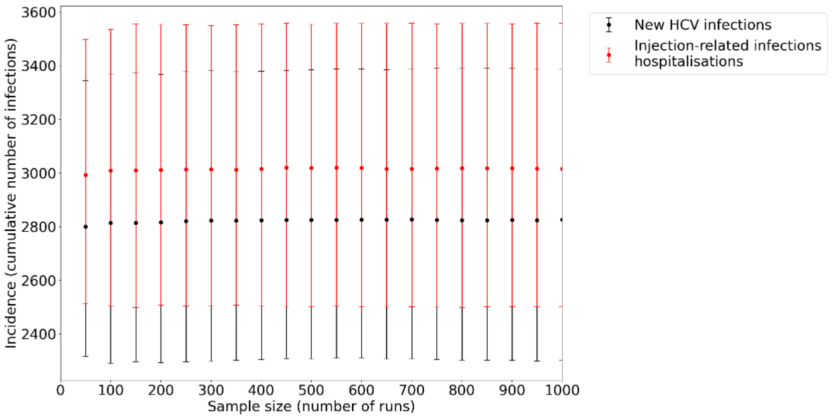

(B) Sample size = 100 runs

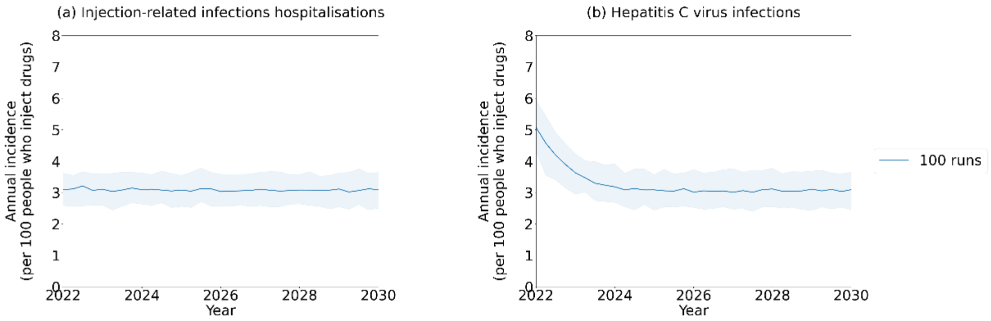

(C) Sample size = 400 runs

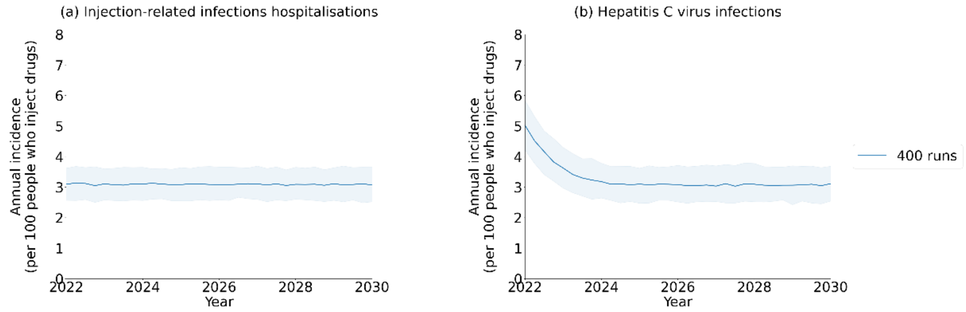

(D) Sample size = 1000 runs

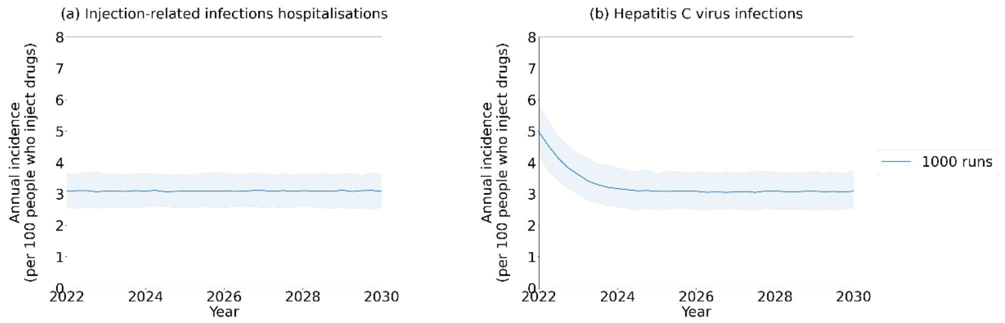

Table 1. Input data for the prison needle and syringe program model in Australia

Parameter	Values	Data sources
Yearly incarcerated population*	2010: 29,700; 2011: 29,107; 2012: 29,380; 2013: 30,773; 2014: 33,789; 2015: 36,134; 2016: 38,845; 2017: 41,202; 2018: 42,974; 2019: 43,028; 2020: 41,060; 2021: 42,970; 2022: 40,591; 2023: 41,929	(2)
Yearly number of prison entrants†	2017: 65,025; 2018: 68,183; 2019: 69,093; 2020: 64,463; 2021: 62,875; 2022: 63,735; 2023: 68,300	(1)
People in prison with injecting drug use history	56%	(3) and Supporting Information, part 2
Probability of injecting drug use continuation within 6 months of incarceration for people in prison with injecting drug use history	0.66	Supporting Information, part 2
Probability of injecting drug use initiation within 6 months of incarceration for people in prison with no injecting drug use history	0.04	Supporting Information, part 2
Chronic HCV infection prevalence among people in prison with injecting drug use history ‡	2015: 51%; 2016: 33%; 2017: 26%; 2018: 20%; 2019: 18%; 2020: 16%; 2021: 16%; 2022: 12%	(4)
Chronic HCV infection prevalence among people in prison with no injecting drug use history	1%	(5)
Incidence of HCV infections per 100 people who inject drugs in prison per year	2014–17: 21.74; 2018–19: 10.25	(3)
Chronic HCV infection prevalence among the entire incarcerated population	2015: 26%; 2016: 32%; 2017: 27%; 2018: 27%; 2019: 24%	(3)
Annual number of HCV treatment initiations in prison	2017: 2,052; 2019: 3,360; 2020: 3,005; 2021: 2,639; 2022: 2,560; 2023: 3,000	2017–22: (6, 7); 2023: Treatment numbers were assumed to return to pre-pandemic levels.
Incidence of hospitalisations for injection-related bacterial or fungal infections per 100 people who inject drugs in prison per year	2013–19: 3.1	(8)
Estimated time spent in prison	7.5 months	Calibrated

* Incarcerated population in Australia as of midnight 30 June for each year. Includes sentenced and unsentenced people.

† Number of receptions into full time custody.

‡ Hepatitis C virus RNA prevalence among people attending needle and syringe programs in the community (6).

2. Probability of injecting drug use during incarceration, based on data from the STOP-C study

Reporting of injecting drug use initiation in prison

Injecting drug use initiation in prison was assessed for all participants with behavioural data at enrolment, irrespective of HCV testing or availability of follow-up data. Of 988 individuals with a history of injecting drug use (injecting drug use) at enrolment, 18% reported injecting drug use initiation in prison.

Median age of injecting drug use initiation by setting:

Community: 17 years; interquartile range (IQR), 15–21 years;

Prison: 21 years; IQR, 19–26 years.

Probability of injecting drug use within 6 months of incarceration

Longitudinal data were available for 1128 people who entered prison during the preceding six months; 631 (56%) reported injecting drugs prior to prison entry, 497 (43%) reported no injecting drug use. Median duration of follow-up was 7 months (IQR, 4-18 months). Median number of previous incarcerations was three (IQR, 1-6). Study visits were pre-scheduled for every 3-6 months, but the interval depended on participant condition. Median time between visits was 5 months (IQR, 3-7 months).

A Markov multistate model was used to assess the probability of transition between injecting and non-injecting behavioural states during incarceration. This method is appropriate giving the varying intervals between visits, varying durations of follow-up, and censoring. Injecting drug use was defined as any injecting drug use in prison during the past six months. It was assumed that any transition between injecting behavioural states was not directly took place between visits. The model was initially fit to the entire population for the full duration of available follow-up. Probability matrices for behavioural transitions were then fit with a set time period of six months. Confidence intervals were estimated using bootstrapping techniques (1000 runs); transitions were drawn with replacement and the probability model repeatedly refitted. Analyses were performed in R 2023.6.0.421 using the *msm* package (https://cran.r-project.org/web/packages/msm/index.html).

Probability of injecting drug use within the first six months of incarceration:

No injecting drug use history: 0.04; 95% confidence interval (CI), 0.03-0.06);

Injecting drug use history: 0.66; 95% CI, 0.62-0.69).

3. Costs of the prison needle and syringe program

Staff estimates

Using information published by the Australian Institute of Health and Welfare, it was estimated that there were twelve full-time equivalent nurses per prison in 2022 (11). The wage of a primary health care nurse was based on the Fair Work Ombudsman pay guide for a registered nurse: level 5, grades 4 (lower bound), 5 (point estimate), and 6 (upper bound) (12).

Prison needle and syringe program kit

The initial kit provided to participants comprises a case, components, and a single dose unit of intranasal naloxone. Subsequent kit exchanges involve components only, while naloxone is replaced at a rate of twelve per 100 participants per year, according to overdose incidence data from the 2023 Australian Needle Syringe Program Survey (13). The lower bound for the frequency of kit exchanges (twice per month) is based on data from the evaluation of pilot prison needle and syringe programs in Switzerland, Germany, and Spain (14). The point estimate and upper bound (four and eight time per month) were obtained by personal communication.

The unit cost of a prison needle and syringe program kit was estimated by obtaining individual components from various suppliers:

Insulin syringes: The kit includes two standard 1mL insulin syringes; they cost $22.80 for a box of 100 from Superior Healthcare, a wholesale supplier for the medical industry and general public throughout Australia (15).

Cotton filters: A pack of five cotton filters are included in the kit; the unit cost is $0.30, from SteriAus, a wholesale supplier to needle and syringe programs throughout Australia (16).

Disinfectant swabs: The inclusion of five disinfectant swabs; $7.50 for a box of 200 from Medshop, an Australian online medical supplies company (17).

Sterile water: The kit contains five plastic ampules of sterile water; $23.90 for a box of 50 from Medshop (18) .

Safecooker: A single safecooker; $0.29 from Steriaus (16).

Plastic container: A plastic container is provided once at enrolment; $3.00 from Kmart Australia (19).

Naloxone spray: A single-dose intranasal naloxone spray; $24.97 according to the Pharmaceutical Benefits Scheme website (20).

The total cost of a single prison needle and syringe program kit, rounded to the nearest integer, was $4.00, excluding the plastic container and naloxone (components only), and $32.00 including these items.

Other costs

Prison needle and syringe program supplies deliveries: It was assumed that prison needle and syringe program supplies are delivered at a frequency of one delivery per month per prison; Australia Post extra-large flat rate packaging: $59.20 (21).

Sharps container: Four-litre sharps container from Medshop: $9.63 (22); and its capacity (number of syringes per container) sourced from ULINE (23).

4. Estimated costs of hospitalisations with injection-related bacterial or fungal infections

Hospital costs

The cost of diagnosing and treating injection-related bacterial or fungal infections in the public sector in Australia was estimated using the data sources and assumptions described below.

Number of events

The number of hospital admissions with injection-related infections was based on the findings of a longitudinal study of people who injected drugs in Melbourne during 2008–2018 (24). The data were organised by infection type: skin or soft tissue infections and invasive infections (bloodstream infection or sepsis, osteomyelitis or septic arthritis, infective endocarditis); information on the median hospital length of stay was also provided. Of 740 hospital admissions with injection-related infections, 490 were uncomplicated skin or soft tissue infections (median length of stay: two days; IQR, 1–4 days); 62 were complicated skin or soft tissue infections (median length of stay: ten days; IQR, 3–28 days); 250 were invasive infections, including 157 cases of bloodstream infection or sepsis (median length of stay: ten days; IQR, 3–24 days); 92 cases of osteomyelitis or septic arthritis (median length of stay: nine days; IQR, 3–25 days); and 80 cases of infective endocarditis (median length of stay: ten days; IQR, 3–26 days).

Cost items

The National Hospital Cost Data Collection Public Hospitals Report by the Independent Health and Aged Care Pricing Authority (IHACPA) (25) provided costs for hospital stays and specific infection treatments (2019–20):

- Cost of managing a bloodstream infection/sepsis (septicaemia): $13,747.91
- Cost of managing osteomyelitis/septic arthritis: $12,155.39
- Cost of managing infective endocarditis: $22,631.76
- Cost per day in hospital: $2,266

The NHCDC cost estimates reflect the resources required to manage specific infection types, including pathology, imaging, ward supplies, pharmacy and critical care.

Key assumptions

- The numbers of admissions for each event type were used as weights in calculations.
- The calculation of the cost per treatment episode from the NHCDC data took into account the numbers of admissions with cases of major, intermediate, and minor complexity.
- The median length of stay and cost per hospital day determined the cost of treating skin or soft tissue infections.
- Lower and upper bounds were based on the lower and upper IQR limits of the length of stay for skin or soft tissue infections hospitalisations.

Mean cost

The mean cost of hospitalisation with injection-related bacterial or fungal infections in a public hospital, converted to 2022–23 dollars and rounded to the nearest dollar was $13,375 (IQR, $10,161–20,666).

Transport costs

The cost of transporting a person from a prison to a hospital was based on non-emergency ambulance fees weighted across states and territories according to their total prison population size (26-33). The estimated cost, $434.09, was added to the mean hospitalisation cost derived above.

Limitations

The cost estimates for hospitalisation with injection-related bacterial or fungal infections are conservative, as they do not take into account costs of security staff required during hospital stays, secure wards at the hospital, or secure transport between facilities.

5. Prison characteristics and security classification

Overview of prisons across Australia

There were 102 public and private prisons in Australia in 2023, excluding 24-hour court cell complexes (table 2). Data sources were the Australian Bureau of Statistics (2) and government websites (34-40).

Table 2. Australian prisons by location and highest security level, 2023 (reference 2, tables 14 to 35)

State/territory	Prison location	Number of prisoners	Highest security level
ACT	Alexander Maconochie Centre	375	Maximum
NSW	Amber Laurel Correctional Centre	12	Minimum
NSW	Bathurst Correctional Centre	637	Maximum
NSW	Bolwara House Transitional Centre	14	Minimum
NSW	Broken Hill Correctional Centre	56	Medium
NSW	Cessnock Correctional Centre	568	Medium
NSW	Clarence Correctional Centre	1180	Maximum
NSW	Compulsory Drug Treatment Correctional Centre	22	Medium
NSW	Cooma Correctional Centre	137	Medium
NSW	Dillwynia Correctional Centre	473	Medium
NSW	Geoffrey Pearce Correctional Centre	319	Minimum
NSW	Glen Innes Correctional Centre	102	Minimum
NSW	Goulburn Correctional Centre	420	Maximum
NSW	High Risk Management Correctional Centre	58	Maximum
NSW	Hunter Correctional Centre	329	Maximum
NSW	John Morony Correctional Centre (I)	421	Medium
NSW	Junee Correctional Centre	881	Medium
NSW	Kariong Correctional Centre	35	Medium
NSW	Kirkconnell Correctional Centre	194	Minimum
NSW	Lithgow Correctional Centre	231	Maximum
NSW	Long Bay Hospital	174	Maximum
NSW	Macquarie Correctional Centre	355	Maximum
NSW	Mannus Correctional Centre	112	Minimum
NSW	Mary Wade Correctional Centre	68	Minimum
NSW	Metropolitan Remand and Reception Centre	911	Maximum
NSW	Metropolitan Special Programs Centre	735	Maximum
NSW	Mid North Coast Correctional Centre	804	Maximum
NSW	Parklea Correctional Centre	1115	Maximum
NSW	Parramatta Transitional Centre	11	Minimum
NSW	Shortland Correctional Centre	514	Maximum
NSW	Silverwater Women's Correctional Centre	147	Maximum
NSW	South Coast Correctional Centre	669	Maximum
NSW	Special Purpose Centre	33	Maximum
NSW	St Heliers Correctional Centre	195	Minimum
NSW	Tamworth Correctional Centre	51	Medium
NSW	Wellington Correctional Centre	320	Maximum
NT	Alice Springs Correctional Centre	666	Maximum
NT	Barkly Work Camp	72	Minimum
NT	Darwin Correctional Centre	1275	Maximum
NT	Darwin Police Prison	41	Maximum
NT	Datjala Work Camp	49	Minimum

State/territory	Prison location	Number of prisoners	Highest security level
QLD	Arthur Gorrie Correctional Centre	1364	Maximum
QLD	Borallon Training and Correctional Centre	814	Maximum
QLD	Brisbane Correctional Centre	887	Maximum
QLD	Brisbane Women's Correctional Centre	314	Maximum
QLD	Capricornia Correctional Centre	1013	Maximum
QLD	Helana Jones Centre	28	Minimum
QLD	Lotus Glen Correctional Centre	1103	Maximum
QLD	Maryborough Correctional Centre	684	Maximum
QLD	Numinbah Correctional Centre - Women's Unit	103	Minimum
QLD	Palen Creek Correctional Centre	199	Minimum
QLD	Southern Queensland Correctional Centre	297	Maximum
QLD	Townsville Correctional Centre	1043	Maximum
QLD	Wolston Correctional Centre	869	Maximum
QLD	Woodford Correctional Centre	1510	Maximum
SA	Adelaide Pre-Release Centre	61	Minimum
SA	Adelaide Remand Centre	256	Maximum
SA	Adelaide Women's Prison	201	Maximum
SA	Cadell Training Centre	185	Minimum
SA	James Nash House	14	Maximum
SA	Mobilong Prison	474	Medium
SA	Mount Gambier Prison	645	Medium
SA	Port Augusta Prison	435	Maximum
SA	Port Lincoln Prison	175	Medium
SA	Yatala Labour Prison	556	Maximum
TAS	Hobart Reception Prison	32	Maximum
TAS	Launceston Reception Prison	27	Maximum
TAS	Mary Hutchinson Women's Prison	43	Maximum
TAS	Risdon Prison Complex	431	Maximum
TAS	Ron Barwick Prison	222	Minimum
VIC	Barwon Prison	292	Maximum
VIC	Beechworth Correctional Centre	142	Minimum
VIC	Dame Phyllis Frost Centre	262	Maximum
VIC	Dhurringile Prison	216	Minimum
VIC	Fulham Correctional Centre	648	Medium
VIC	Hopkins Correctional Centre	619	Medium
VIC	Judy Lazarus Transition Centre	15	Minimum
VIC	Langi Kal Kal Prison	370	Minimum
VIC	Loddon Prison	523	Medium
VIC	Marngoneet Correctional Centre	619	Medium
VIC	Melbourne Assessment Prison	174	Maximum
VIC	Metropolitan Remand Centre	744	Maximum
VIC	Port Phillip Prison	835	Maximum
VIC	Ravenhall Correctional Centre	938	Medium
VIC	Tarrengower Prison	39	Minimum
WA	Acacia Prison	1298	Medium
WA	Albany Regional Prison	396	Maximum
WA	Bandyup Women's Prison	225	Maximum
WA	Boronia Pre-release Centre for Women	70	Minimum
WA	Broome Regional Prison	61	Minimum

State/territory	Prison location	Number of prisoners	Highest security level
WA	Bunbury Regional Prison	508	Maximum
WA	Casuarina Prison	1191	Maximum
WA	Eastern Goldfields Regional Prison	237	Minimum
WA	Greenough Regional Prison	234	Maximum
WA	Hakea Prison	991	Maximum
WA	Karnet Prison Farm	323	Minimum
WA	Melaleuca Women's Prison	208	Maximum
WA	Pardelup Prison Farm	86	Minimum
WA	Roebourne Regional Prison	213	Maximum
WA	Wandoo Rehabilitation Prison	50	Medium
WA	West Kimberley Regional Prison	213	Medium
WA	Wooroloo Prison Farm	400	Minimum

Sensitivity analyses: security scenarios

Recognising that introducing needle and syringe programs in all prisons might not be feasible or likely, we modelled security scenarios in which only the programs were introduced only in some prisons. These scenarios assumed the same needle and syringe program coverage as the main model, but it was selectively applied to prisons by security levels (minimum only, medium only, or maximum only). Prisons were classified according to their highest security level:

- Maximum security: 54 prisons (70% of prisoners).
- Medium security: 20 prisons (21% of prisoners).
- Minimum security: 28 prisons (9% of prisoners).

Given the lack of information about differences in epidemiological and behavioural parameters between prisons of different security levels, these scenarios were included in the main model by varying the levels of prison needle and syringe program coverage based on the proportion of prisoners in each security category. The costs were then adjusted to reflect the number of prisons included in the security scenario. For example, for the 28 minimum security prisons, it was assumed that by 2030 the prison needle and syringe program would include 50% of people who inject drugs, and the cost calculations included only the 28 prisons in the minimum security category. The operation model of the prison needle and syringe program was assumed to be independent of prison security level.

6. Effectiveness of the prison needle and syringe program

As the prison needle and syringe program is prospective, the direct impact of the program on the incidence of HCV and injection-related bacterial or fungal infections can only be estimated (it could be evaluated in a pilot program). Three relationships must be considered: the relationship between prison needle and syringe program coverage (proportion of people who inject drugs in prison who use the service) and the reduction in needle and equipment sharing events; the relationship between the reduction in needle and equipment sharing events and the reduction in HCV infection risk; and the relationship between prison needle and syringe program coverage and the reduction in injection-related bacterial or fungal infection risk.

Relationship between prison needle and syringe program coverage and reduction in sharing events

Estimates from studies of community needle and syringe programs indicate that 5-19% of people in the programs continue to share equipment (41, 42); however, the reasons that people continue to share equipment in the community are likely to be very different from those in prisons. In the main analysis, we assume that people no longer share equipment while participating in the prison needle and syringe program; in sensitivity analyses, we assess the effect of 5% or 19% of people continuing to share equipment while participating in the program. A compartmental model does not assign individuals to permanently using the program, but instead considers a set number of people use the program at a given point in time (ie, individuals can leave or return to the program).

Relationship between reduction in sharing events and HCV transmission risk

Not all sharing events entail the same risk of HCV transmission, because risk varies according to injecting network and other injecting characteristics. For example, if people using the prison needle and syringe program are in more connected or higher risk injecting networks, program coverage (and the subsequent reduction in sharing events) may result in a disproportional reduction in HCV infection risk (eg, 50% coverage could reduce risk by more than 50%). Conversely, if people using the program are initially more risk-adverse, greater program coverage may achieve a smaller reduction in risk. For our main analysis, we assumed a linear relationship between reduction in sharing events and reduction in risk; in sensitivity analyses, we assessed outcomes when the program disproportionately prevented higher or lower risk events.

Relationship between prison needle and syringe program coverage and injection-related bacterial or fungal infection risk

Estimates based on community studies indicate that frequent participation inf needle and syringe programs can result in a 62% reduction in hospitalisations with injection-related bacterial or fungal infections (8). In the main analysis, we assumed a linear relationship between coverage and the reduction in injection-related infection risk (ie, 50% coverage leads to 50% × 62% = 31% reduction in number of hospitalisations). As injection-related infection risk varies according to injecting characteristics, in sensitivity analyses, we assessed outcomes when the program disproportionately prevented higher or lower risk events.

Supplementary results

Table 3. Sensitivity analysis results for the prison needle and syringe program model in Australia, showing the impact of varying cost parameters, model input parameters and prison needle and syringe program coverage on the costs, benefits, and benefit–cost ratio*

			Costs		Benefits		Benefit–cost ratio	
**Scenario**	**HCV infections averted**	**Injection-related bacterial or fungal infection hospitalisations averted**	**$ million**	**Change***	**$ million**	**Change***	**Value**	**Change***
**Prison needle and syringe program scale-up scenario (baseline)**	**894**	**522**	**12.2**	**-**	**31.7**	**-**	**2.6**	**-**
Program coverage of people who inject drugs in prison: 25% v 50% (LB)	465	259	6.3	-48%	16.4	-48%	2.6	0%
Program coverage of people who inject drugs in prison: 75% v 50% (UB)	1,287	779	18.1	48%	46.1	45%	2.5	-2%
Percentage of prisons with a needle and syringe program: 50% v 100% (LB)	465	259	6.1	-50%	16.4	-48%	2.7	3%
Receptive sharing of a needle or syringe: 5% v 0% (LB)	852	496	12.2	0%	30.2	-5%	2.5	-5%
Receptive sharing of a needle or syringe: 19% v 0% (UB)	734	420	12.2	0%	26.0	-18%	2.1	-18%
Reduction in risk events: Lower risk events v Main analysis (LB)	358	201	12.2	0%	12.4	-61%	1.0	-61%
Reduction in risk events: Higher risk events v Main analysis (UB)	1,492	903	12.2	0%	54.2	71%	4.4	71%
Program rollout period: 1 year v 2 years (LB)	482	269	6.3	-48%	17.1	-46%	2.7	4%
Program rollout period: 4 years v 2 years (UB)	406	231	5.4	-56%	14.2	-55%	2.7	2%
Discounting: 2.5% v 5.0% (LB)	894	522	13.9	14%	36.3	14%	2.6	0%
Discounting: 10.0% v 5.0% (UB)	894	522	9.6	-22%	24.6	-23%	2.6	-1%
Benefits: HCV infections averted only v HCV infections + Injection-related infections averted	894	0	12.2	0%	26.3	-17%	2.2	-17%
Wage of primary health care nurse: $52.48 v $57.88 (LB)	894	522	11.7	-5%	31.7	0%	2.7	5%
Wage of primary health care nurse: $63.33 v $57.88 (UB)	894	522	12.8	5%	31.7	0%	2.5	-4%

			Costs		Benefits		Benefit–cost ratio	
**Scenario**	**HCV infections averted**	**Injection-related bacterial or fungal infection hospitalisations averted**	**$ million**	**Change***	**$ million**	**Change***	**Value**	**Change***
Number of kit exchanges per participant per month: 2 v 4 (LB)	894	522	7.2	-41%	31.7	0%	4.4	69%
Number of kit exchanges per participant per month: 8 v 4 (UB)	894	522	22.2	82%	31.7	0%	1.4	-45%
Cost of HCV treatment: $5,000 v $36,111 (LB)	894	522	12.2	0%	10.5	-67%	0.9	-67%
Cost of HCV treatment: $15,000 v $36,111 (UB)	894	522	12.2	0%	17.5	-45%	1.4	-45%
Cost of injection-related infection hospitalisation: $10,595 v $13,809 (LB)	894	522	12.2	0%	30.5	-4%	2.5	-4%
Cost of injection-related infection hospitalisation: $21,100 v $13,809 (UB)	894	522	12.2	0%	34.6	9%	2.8	9%
Incidence of injection-related infection hospitalisations (initial): 1.6 v 3.1 per 100 people who inject drugs per year (LB)	894	258	12.2	0%	29.0	-9%	2.4	-9%
Incidence of injection-related infection hospitalisations (initial): 6.2 v 3.1 per 100 people who inject drugs per year (UB)	894	167	12.2	0%	28.0	-12%	2.3	-12%
Incidence of HCV infections(initial): 10.9 v 21.7 per 100 people who inject drugs per year (LB)	356	522	12.2	0%	15.9	-50%	1.3	-50%
Incidence of HCV infections (initial): 43.5 v 21.7 per 100 people who inject drugs per year (UB)	3,018	522	12.2	0%	94.4	197%	7.7	198%
Chronic HCV prevalence among people with injecting drug use history (2025): 6% v 12% (LB)	389	522	12.3	1%	16.8	-47%	1.4	-47%
Chronic HCV prevalence among people with injecting drug use history (2025): 24% v 12% (UB)	2,555	522	12.2	0%	81.8	158%	6.7	158%
HCV treatment uptake in prison (from 2025): 1,500 v 3,000 (LB)	1,563	522	12.2	0%	52.2	64%	4.3	64%
HCV treatment uptake in prison (from 2025): 6,000 v 3,000 (UB)	753	522	12.4	2%	27.4	-14%	2.2	-15%
People in prison with injecting drug use history: 28% v 56% (LB)	426	282	7.0	-42%	15.4	-52%	2.2	-16%
People in prison with injecting drug use history: 100% v 56% (UB)	2,349	886	20.5	68%	79.4	150%	3.9	49%

			Costs		Benefits		Benefit–cost ratio	
**Scenario**	**HCV infections averted**	**Injection-related bacterial or fungal infection hospitalisations averted**	**$ million**	**Change***	**$ million**	**Change***	**Value**	**Change***
Probability of injecting drug use continuation in prison: 0.33 v 0.66 (LB)	427	274	6.6	-46%	15.4	-51%	2.3	-10%
Probability of injecting drug use continuation in prison: 1.00 v 0.66 (UB)	1,456	776	18.0	47%	51.0	61%	2.8	9%
Probability of injecting drug use initiation in prison: 0.02 v 0.04 (LB)	872	514	12.0	-2%	31.0	-2%	2.6	0%
Probability of injecting drug use initiation in prison: 0.08 v 0.04 (UB)	948	547	12.8	4%	33.6	6%	2.6	1%
Estimated time spent in prison: 0.3 years v 0.6 years (LB)	853	256	7.3	-40%	28.0	-12%	3.8	47%
Estimated time spent in prison: 1.3 years v 0.6 years (UB)	1,862	1,034	22.2	82%	65.8	107%	3.0	14%
Reduction in people who inject drugs in prison (from 2025): 20% v Status-quo (LB)	686	425	9.9	-19%	24.6	-22%	2.5	-4%
Reduction in people who inject drugs in prison (from 2025): 60% v Status-quo (UB)	320	211	5.2	-58%	11.6	-63%	2.2	-14%
Chronic HCV prevalence among people with injecting drug use history (2025): 4% v 12%	273	522	12.3	1%	13.4	-58%	1.1	-58%
Chronic HCV prevalence among people with injecting drug use history (2025): 3% v 12%	199	522	12.3	1%	11.2	-65%	0.9	-65%
Chronic HCV prevalence among people with injecting drug use history (2025): 2% v 12%	136	522	12.3	1%	9.4	-70%	0.8	-71%
Chronic HCV prevalence among people with injecting drug use history (2025): 1% v 12%	68	522	12.3	0%	7.4	-77%	0.6	-77%

* The proportional changes are relative to main model. Only median values are shown.

HCV: Hepatitis C virus; UB: Upper bound; LB: Lower bound.

Table 4. Sensitivity analysis: impact of prison needle and syringe programs in prisons of specific security levels

			Costs		Benefits		Benefit to cost ratio	
**Scenario**	**HCV infections averted**	**Injection-related bacterial or fungal infection hospitalisations averted**	**$ million**	**Change***	**$ million**	**Change***	**Value**	**Change***
**Prison needle and syringe program scale-up scenario (baseline)**	**894**	**522**	**12.2**	**-**	**31.7**	**-**	**2.6**	**-**
Security scenario: minimum security prisons vs. all prisons	85	49	1.2	-90%	3.0	-91%	2.5	-6%
Security scenario: medium security prisons vs. all prisons	201	109	2.6	-79%	7.0	-78%	2.7	5%
Security scenario: maximum security prisons vs. all prisons	645	365	8.4	-31%	22.8	-28%	2.7	4%

Figure 5. Modelled relationship between prison needle and syringe program coverage and reduction in hepatitis C virus (HCV) transmission risk. This depends on (1) the relationship between prison needle and syringe program coverage and reduction in needle and equipment sharing; and (2) the relationship between reduction in sharing and reduction in transmission risk**.** As the prison needle and syringe program is prospective, empirical data is not available and a linear relationship is assumed in the main analysis, and sensitivity analyses assess alternatives.

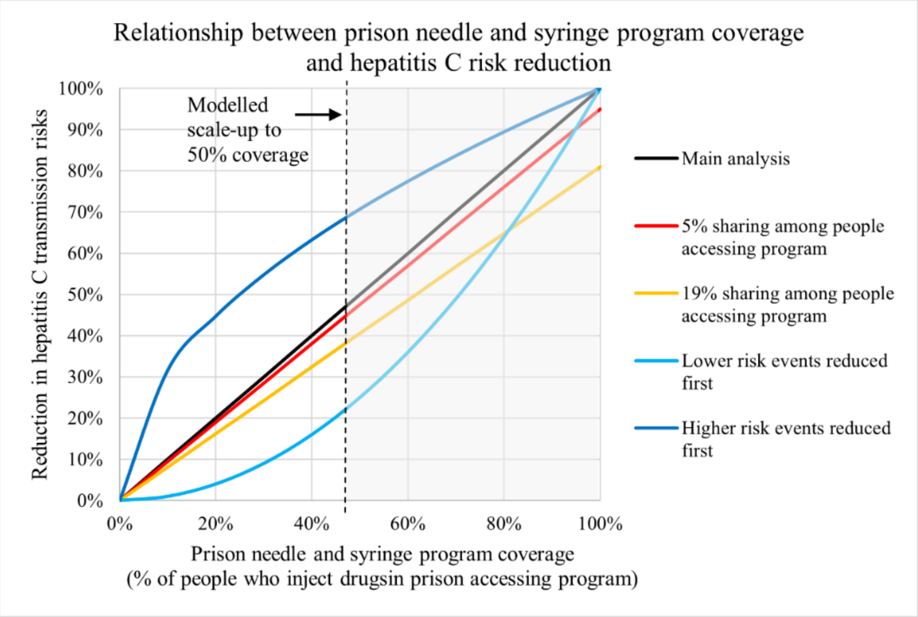

Figure 6. Modelled relationship between prison needle and syringe program coverage and reduction in injection-related bacterial or fungal infection risks. As the prison needle and syringe program is prospective, empirical data is not available, a linear relationship is assumed in the main analysis; sensitivity analyses assess alternatives.

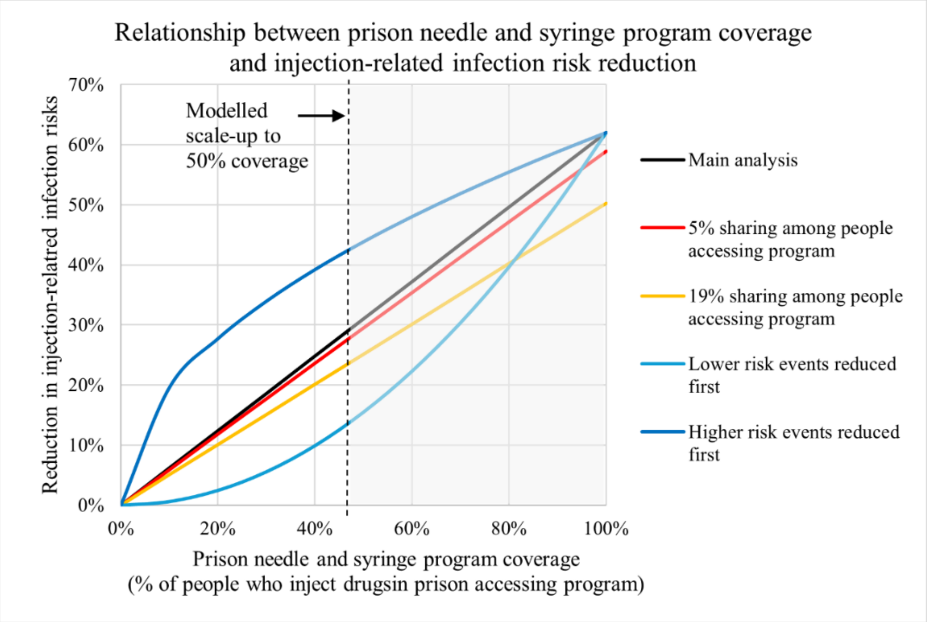

Acknowledgments: The STOP-C Study group

The SToP-C study was a partnership project involving the Kirby Institute, Justice Health and Forensic Mental Health Network, Corrective Services NSW, NSW Ministry of Health, NSW Users and AIDS Association, Hepatitis NSW, and Gilead Sciences.

Protocol Steering Committee: Stuart Loveday (Chair, Hepatitis NSW); Gregory Dore, Andrew Lloyd, Jason Grebely, Tony Butler, Georgina Chambers, Carla Treloar, and Marianne Byrne (UNSW Sydney); Roy Donnelly, Colette McGrath, Julia Bowman, Lee Trevethan, and Katerina Lagios (Justice Health & Forensic Mental Health Network); Luke Grant, and Terry Murrell (Corrective Services NSW); Nicky Bath, Victor Tawil, Annabelle Stevens, and Libby Topp (NSW Health); Alison Churchill, and Kate Pinnock (Community Restorative Centre); Natasha Martin (University of California San Diego); Steven Drew (Hepatitis NSW); Mary Harrod (NSW Users and AIDS Association)

Coordinating Centre (The Kirby Institute, UNSW Sydney): Gregory Dore, Andrew Lloyd, Behzad Hajarizadeh, Lise Lafferty, Tony Butler, Pip Marks, Mahshid Tamaddoni, Stephanie Obeid, Gerard Estivill Mercade, Maria Martinez, and Marianne Byrne

Laboratory Services (NSW Health Pathology): William Rawlinson, Malinna Yeang, Matthew Wynn, and Christiana Willenborg

Site Research Co-ordinators: Angela Smith, Ronella Williams, Brigid Cooper, Kelly Somes, Carina Burns, Camilla Lobo, Karen Conroy, Luke McCredie, Carolyn Café, and Jodie Anlezark.

References

1. Australian Bureau of Statistics (Reference period: December Quarter 2023). Corrective Services, Australia. https://www.abs.gov.au/statistics/people/crime-and-justice/corrective-services-australia/dec-quarter-2023 (accessed 2024 Apr).

2. Australian Bureau of Statistics (Reference period: 2023). Prisoners in Australia. https://www.abs.gov.au/statistics/people/crime-and-justice/prisoners-australia/2023 (accessed 2024 Mar).

3. Hajarizadeh B, Grebely J, Byrne M, et al. Evaluation of hepatitis C treatment-as-prevention within Australian prisons (SToP-C): a prospective cohort study. *Lancet Gastroenterol Hepatol* 2021; 6: 533–546.

4. King J, McManus H, Kwon J, et al. HIV, viral hepatitis and sexually transmissible infections in Australia: Annual surveillance report. Sydney: Kirby Institute, UNSW Sydney, 2023. https://www.kirby.unsw.edu.au/research/reports/asr2023 (accessed Mar 2024).

5. Kirby Institute. National Prison Entrants' Bloodborne Virus and Risk Behaviour Survey Report 2004, 2007, 2010, 2013 and 2016. Sydney: UNSW, 2017. https://www.kirby.unsw.edu.au/sites/default/files/documents/JHP_National-Prison-Entrants-Report-2004-2007-2010-2013.pdf (accessed Jan 2024).

6. Burnet Institute and Kirby Institute. Australia's progress towards hepatitis C elimination: annual report 2023. Melbourne: Burnet Institute, 2023. https://www.burnet.edu.au/media/tahd41iz/australias-progress-towards-hepatitis-c-elimination-annual-report-2023.pdf (accessed Jan 2024).

7. Papaluca T, Hellard ME, Thompson AJV, Lloyd AR. Scale-up of hepatitis C treatment in prisons is key to national elimination. *Med J Aust* 2019; 210: 391–393.e1.

8. Brothers TD, Lewer D, Jones N, et al. Effect of incarceration and opioid agonist treatment transitions on risk of hospitalisation with injection drug use-associated bacterial infections: A self-controlled case series in New South Wales, Australia. *Int J Drug Policy* 2023; 122: 104218.

9. Dunleavy K, Munro A, Roy K, et al. Association between harm reduction intervention uptake and skin and soft tissue infections among people who inject drugs. *Drug Alcohol Depend* 2017; 174: 91–97.

10. Robert LG. Converting an odds ratio to a range of plausible relative risks for better communication of research findings. *BMJ* 2014; 348: f7450.

11. Australian Institute of Health and Welfare. The health of people in Australia's prisons 2022, catalogue number PHE 334. Canberra: AIHW, 2023. https://www.aihw.gov.au/getmedia/e2245d01-07d1-4b8d-81b3-60d14fbf007f/aihw-phe-33-health-of-people-in-australias-prisons-2022.pdf (accessed 2024 Jan).

12. Fairwork Ombudsman. Pay Guide Nurses Award 2023; Registered nurse - level 5, grade 6. Commonwealth of Australia, 2023. https://library.fairwork.gov.au/award/?krn=MA000034&awardid=da2fa0bf-5488-ee11-be36-6045bde4a990 (accessed 2024 Jan).

13. Kirby Institute. Australian NSP survey national data report 2018 - 2022. Sydney: UNSW, 2023. https://www.kirby.unsw.edu.au/sites/default/files/documents/ANSPS_National-Data-Report-2018-2022.pdf (accessed 2024 Jan).

14. Stöver H, Nelles J. Ten years of experience with needle and syringe exchange programmes in European prisons. *Int J Drug Policy* 2003; 14: 437–444.

15. Superior Healthcare. Insulin Syringe Standard 1mL 27gx8mm BOX 100: Superior Healthcare. https://www.superiorhealthcare.com.au/GMMD020_dash_100/Insulin-Syringe-Standard-1mL-27gx8mm-BOX-100/pd.php (accessed 2023 Dec).

16. SteriAus. Ordering | Health Professionals: SteriAus. https://steriaus.com.au/ordering/ (accessed 2023 Dec).

17. Medshop. BSN Medical Medi-Swab Alcohol Swabs Medshop. https://www.medshop.com.au/products/bsn-medical-medi-swabs (accessed 2023 Dec).

18. Medshop. Pfizer Water For Injection Ampules x50: Medshop. https://www.medshop.com.au/products/water-for-inj-10ml-ster-amp-x50-water-for-injection-amp (accessed 2023 Dec).

19. Kmart. 1.1L Clip Container. https://www.kmart.com.au/product/11l-clip-container-42919520/ (accessed 2023 Dec).

20. The Pharmaceutical Benefits Scheme. Naloxone. Canberra: Dept. of Health and Aged Care, 2023. https://www.pbs.gov.au/medicine/item/11816X-11817Y (accessed 2023 Dec).

21. Australia Post. Extra Large Flat Rate Packaging. https://auspost.com.au/shop/sending/flat-rate-packaging/extra-large (accessed 2023 Dec).

22. Medshop. Sharps Container - 4L. https://www.medshop.com.au/products/sharps-container?Size=4L (accessed 2024 Mar).

23. ULINE. Sharps Container - 5 Quart. https://www.uline.com/Product/Detail/S-15308/First-Aid/Sharps-Container-5-Quart (accessed 2024 Mar).

24. Curtis SJ, Colledge-Frisby S, Stewardson AJ, et al. Prevalence and incidence of emergency department presentations and hospital separations with injecting-related infections in a longitudinal cohort of people who inject drugs. *Epidemiol Infect* 2023; 151: e192.

25. Independent Health and Aged Care Pricing Authority (IHACPA). National Hospital Cost Data Collection (NHCDC) Public Sector Cost Report 2019-20. Canberra: Department of Health and Aged Care, 2022. https://www.ihacpa.gov.au/sites/default/files/2022-08/NHCDC%20Round%2024%20Appendix.xlsm (accessed 2024 Jan).

26. NSW Ambulance. Accounts & Fees: NSW Government. https://www.ambulance.nsw.gov.au/our-services/accounts-and-fees (accessed 2024 Sep).

27. Tasmanian Legislation. Ambulance Service (Fees) Regulations 2021 Statutory Rules 2021, No. 69: Tasmanian Government. https://www.legislation.tas.gov.au/view/pdf/asmade/sr-2021-069 (accessed 2024 Sep).

28. Department of Health, Victoria. Ambulance fees. https://www.health.vic.gov.au/patient-care/ambulance-fees (accessed 2024 Sep).

29. Queensland Government. Queensland Ambulance Treatment and Transport. https://www.qld.gov.au/emergency/emergencies-services/qld-visitors-qas (accessed 2024 Sep).

30. St John WA. Ambulance fees. https://stjohnwa.com.au/ambulance-and-health-services/metro-ambulance-service/metro-ambulance-fees (accessed 2024 Sep).

31. Service SA. Fees and charges: SA Health. https://saambulance.sa.gov.au/what-we-do/fees-and-charges/ (accessed 2024 Sep).

32. ACT Emergency Services Agency. Fees and charges. https://esa.act.gov.au/about-esa-emergency-services/ambulance/fees-and-charges (accessed 2024 Sep).

33. St John NT. Ambulance Transport Rates 2023. https://www.stjohnnt.org.au/img/documents/ambulance/Ambulance%20Transport%20Rates%201%20Feb%202023.pdf (accessed 2024 Sep).

34. ACT Corrective Services. Australian Capital Territory Government, Justice and Community Safety Directorate: Alexander Maconochie Centre, 2024. https://www.correctiveservices.act.gov.au/custody/alexander-maconochie-centre (accessed 2024 Apr).

35. Corrections Victora. Prisons in Victoria. https://www.corrections.vic.gov.au/contact-or-visit-a-prison/prisons-in-victoria (accessed 2024 Sep).

36. Corrective Services NSW. Correctional centres. https://correctiveservices.dcj.nsw.gov.au/correctional-centres.html (accessed 2024 Mar).

37. Department for Correctional Services. Prison locations. https://www.corrections.sa.gov.au/prison/prison-locations (accessed 2024 Mar).

38. Corrective Services. Corrective Services: Government of Western Australia, Department of Justice, 2024. https://www.wa.gov.au/organisation/department-of-justice/corrective-services (accessed 2024 Mar).

39. Northern Territory Government information and services. Prisons and probation. https://nt.gov.au/law/prisons (accessed 2024 Mar).

40. Queensland Corrective Services. Prison locations. https://www.qld.gov.au/law/sentencing-prisons-and-probation/prisons-and-detention-centres/prison-locations (accessed 2024 Mar).

41. Heard S, Maher L. Australian Needle Syringe Program Survey National Data Report 2019-2023: Prevalence of HIV, HCV and injecting and sexual behaviour among NSP attendees. Sydney: Kirby Institute, UNSW Sydney, 2024. https://www.kirby.unsw.edu.au/sites/default/files/documents/Australian-NSP-Survey-national-data-report_2019-2023.pdf (accessed Sep 2024).

42. Sutherland R, Uporova J, King C, et al. Australian Drug Trends 2023: Key Findings from the National Illicit Drug Reporting System (IDRS) Interviews. Sydney: National Drug and Alcohol Research Centre, UNSW Sydney, 2023. https://ndarc.med.unsw.edu.au/sites/default/files/ndarc/resources/National_IDRS_2023_Report_Final%5B1%5D.pdf (accessed Sep 2024).

CHEERS 2022 Checklist

Topic	No.	Item	Location where item is reported
**Title**			
	1	Identify the study as an economic evaluation and specify the interventions being compared.	Title page, page 1
**Abstract**			
	2	Provide a structured summary that highlights context, key methods, results, and alternative analyses.	Page 2
**Introduction**			
**Background and objectives**	3	Give the context for the study, the study question, and its practical relevance for decision making in policy or practice.	Introduction
**Methods**			
**Health economic analysis plan**	4	Indicate whether a health economic analysis plan was developed and where available.	Methods, Section "Cost-benefit analysis"
**Study population**	5	Describe characteristics of the study population (such as age range, demographics, socioeconomic, or clinical characteristics).	Methods, Section "Prison model", Subsections “Model overview”
**Setting and location**	6	Provide relevant contextual information that may influence findings.	Methods, Section "Prison model"
**Comparators**	7	Describe the interventions or strategies being compared and why chosen.	Methods, Section "Model-based analysis", Subsection "Scenarios projected"
**Perspective**	8	State the perspective(s) adopted by the study and why chosen.	Methods, Section "Cost-benefit analysis"
**Time horizon**	9	State the time horizon for the study and why appropriate.	Methods, Section "Prison model", Subsections “Model overview” and Section "Model-based analysis", Subsection "Scenarios projected"
**Discount rate**	10	Report the discount rate(s) and reason chosen.	Methods, Section "Cost-benefit analysis"
**Selection of outcomes**	11	Describe what outcomes were used as the measure(s) of benefit(s) and harm(s).	Methods, Section "Cost-benefit analysis", Subsections "Costs" and “Benefits”
**Measurement of outcomes**	12	Describe how outcomes used to capture benefit(s) and harm(s) were measured.	Methods, Section "Model-based analysis", Subsection " Epidemiological outcome measures" and Section "Cost-benefit analysis", Subsection "Benefit-cost ratio"
**Valuation of outcomes**	13	Describe the population and methods used to measure and value outcomes.	Methods, Section "Cost-benefit analysis", Subsection "Benefit-cost ratio"
**Measurement and valuation of resources and costs**	14	Describe how costs were valued.	Methods, Section "Cost-benefit analysis", Subsections "Costs" and "Benefits"
**Currency, price date, and conversion**	15	Report the dates of the estimated resource quantities and unit costs, plus the currency and year of conversion.	Methods, Section "Cost-benefit analysis", Subsections "Costs" and "Benefits"
**Rationale and description of model**	16	If modelling is used, describe in detail and why used. Report if the model is publicly available and where it can be accessed.	Methods, Section "Model overview" and Appendix A
**Analytics and assumptions**	17	Describe any methods for analysing or statistically transforming data, any extrapolation methods, and approaches for validating any model used.	Methods, Section "Model-based analysis", and Section "Model-based analysis", Subsection " Epidemiological outcome measures" and Section "Cost-benefit analysis", Subsection "Benefit-cost ratio"
**Characterising heterogeneity**	18	Describe any methods used for estimating how the results of the study vary for subgroups.	Not Applicable
**Characterising distributional effects**	19	Describe how impacts are distributed across different individuals or adjustments made to reflect priority populations.	Not Applicable

Topic	No.	Item	Location where item is reported
**Characterising uncertainty**	20	Describe methods to characterise any sources of uncertainty in the analysis.	Methods, Section "Model-based analysis", Subsection " Epidemiological outcome measures" and Section "Cost-benefit analysis", Subsection "Benefit-cost ratio"
**Approach to engagement with patients and others affected by the study**	21	Describe any approaches to engage patients or service recipients, the general public, communities, or stakeholders (such as clinicians or payers) in the design of the study.	Not Applicable
**Results**			
**Study parameters**	22	Report all analytic inputs (such as values, ranges, references) including uncertainty or distributional assumptions.	Methods, Section "Model overvier", and Tables 1, 2 and 3
**Summary of main results**	23	Report the mean values for the main categories of costs and outcomes of interest and summarise them in the most appropriate overall measure.	Results, Section "The status-quo and PNSP scale-up scenarios"
**Effect of uncertainty**	24	Describe how uncertainty about analytic judgments, inputs, or projections affect findings. Report the effect of choice of discount rate and time horizon, if applicable.	Results, Section "Sensitivity analyses"
**Effect of engagement with patients and others affected by the study**	25	Report on any difference patient/service recipient, general public, community, or stakeholder involvement made to the approach or findings of the study	Not Applicable
**Discussion**			
**Study findings, limitations, generalisability, and current knowledge**	26	Report key findings, limitations, ethical or equity considerations not captured, and how these could affect patients, policy, or practice.	Discussion
**Other relevant information**			
**Source of funding**	27	Describe how the study was funded and any role of the funder in the identification, design, conduct, and reporting of the analysis	End of manuscript, "Primary funding"
**Conflicts of interest**	28	Report authors conflicts of interest according to journal or International Committee of Medical Journal Editors requirements.	End of manuscript, "Competing interests"

From: Husereau D, Drummond M, Augustovski F, et al. Consolidated Health Economic Evaluation Reporting Standards 2022 (CHEERS 2022) Explanation and Elaboration: A Report of the ISPOR CHEERS II Good Practices Task Force. Value Health 2022;25. doi:10.1016/j.jval.2021.10.008
